# Informal caregivers' perceptions of oral care and their association with the use of oral health services: A cross‐sectional study among informal caregivers and their care recipients

**DOI:** 10.1002/cre2.552

**Published:** 2022-04-03

**Authors:** Karoliina Holmavuo, Anna Liisa Suominen, Johanna Lammintakanen, Irma Nykänen, Tarja Välimäki, Sohvi Koponen, Roosa‐Maria Savela, Ursula Schwab

**Affiliations:** ^1^ Department of Health and Social Management, Faculty of Social Sciences and Business Studies University of Eastern Finland Kuopio Finland; ^2^ Institute of Dentistry University of Eastern Finland Kuopio Finland; ^3^ Department of Oral and Maxillofacial Diseases Kuopio University Hospital Kuopio Finland; ^4^ Kuopio Research Centre of Geriatric Care, School of Pharmacy, Faculty of Health Sciences University of Eastern Finland; ^5^ Institute of Public Health and Clinical Nutrition, Faculty of Health Sciences University of Eastern Finland Kuopio Finland; ^6^ Department of Nursing Science, Faculty of Health Sciences University of Eastern Finland Kuopio Finland; ^7^ Department of Endocrinology and Clinical Nutrition Kuopio University Hospital Kuopio Finland

**Keywords:** informal caregivers, oral care, oral health services, perceptions

## Abstract

**Objective:**

This study aimed to describe informal caregivers' perceptions of the importance of oral care and investigate the association between these perceptions and the use of oral health services during the past year.

**Background:**

There is limited research on informal caregivers' perceptions of oral care. These perceptions presumably influence oral self‐care along with caregivers' and care recipients' use of oral health services.

**Materials and Methods:**

Baseline data from the multidisciplinary Lifestyle, Nutrition, and Oral health in caregivers (LENTO) intervention study were analyzed. Informal caregivers (*n* = 125) and care recipients (*n* = 120) ≥65 years of age and living in Eastern Finland participated in the study. Data were collected through semi‐structured interviews.

**Results:**

A majority (81%) of the informal caregivers considered oral care very important. Informal caregivers who considered oral care very important had 10 or more years of education, and considered service fees reasonable were more likely to have visited oral health services during the past year than other caregivers. No association between informal caregivers' perceptions of oral care and care recipients' use of oral health services during the past year was observed.

**Conclusions:**

The study provides insight into informal caregivers' perceptions of oral care, with most informal caregivers considering oral care to be very important. Our findings support what has been reported in previous studies in that favorable perceptions of oral care are associated with oral health service visits. This association, however, did not hold true for care recipients' use of services.

## 1 INTRODUCTION

A previous World Health Organization report on aging and health (2015) notes that oral health, which is crucial for healthy aging, often receives too little attention (Ogawa & Petersen, 2015). Poor oral health can predispose individuals to pain, infections, and loss of teeth. There is also strong evidence that the presence of oral disease is associated with the occurrence of several other diseases, for example, type 2 diabetes and cardiovascular and respiratory diseases (Liljestrand et al., 2015). In addition, the association between oral health and nutrition exerts a significant effect on an individual's quality of life (Henshaw & Calabrese, 2001; Palmer, 2001). Unfortunately, there is evidence that older people have worse oral health and hygiene than younger people (Nihtilä et al., 2017). The most important elements to maintaining oral health are appropriate oral self‐care and regular use of oral health services (Suominen & Raittio, 2018). Impaired functional or cognitive capacity can act as a risk factor to poor oral hygiene and health, and might also complicate the use of oral health services (Coll et al., 2020). In contrast, the most common barriers to accessing oral health services among older people are impaired mobility and cognitive function (Komulainen et al., 2012), multimorbidity, poor access to oral health services (Nitschke et al., 2001), and high cost of care (Lo & Schwarz, 1994).

An aim of Finnish social welfare is to support older people living at home for as long as possible (Finnish Ministry of Social Affairs and Health, 2014). However, older people living at home need help to cope with daily living activities and can receive either informal or formal (professional) assistance. Informal care can be considered as part of the interface between informal and formal assistance (Blomgren et al., 2006). The current practice for arranging informal care in Finland is to make a contract (i.e., informal care agreement) between the municipality and the informal caregiver (IC) when the IC and care recipient (CR) meet specific function or health‐related criteria (Government of Finland, 2005; Ring, 2021). In Finland, the Act on Support for Informal Care (937/2005) requires municipalities may grant services to CRs and care allowance, leave and support services to ICs. The municipality must arrange, if necessary, welfare and health check‐ups for the IC, as well as social‐ and health care services that support their ability to provide care. (Government of Finland, 2005) However, it is important to state that—by the Finnish Employment Contracts Act—ICs are not employed by the municipality; instead, the contract is a mandate agreement (Ring, 2021). An increasing number (57%) (Finnish Institute of Health and Welfare, 2021) of Finnish ICs with an informal care agreement were over 65 years in 2019. According to 2017 statistics from the Finnish Institute of Health and Welfare, the majority (70%) of ICs with an informal care agreement were females and, in most cases, the CR was their spouse (Noro et al., 2018).

Favorable attitudes and perceptions toward oral care have been found to improve oral health in terms of less toothache among adults (Edman et al., 2018) and older people (Gilbert et al., 1993) and increase the frequency of preventive dental visits among adults (Edman et al., 2018; Riley et al., 2006) and older people (Gilbert et al., 1994; Tennstedt et al., 1994). However, research on how ICs perceive oral care is very limited. Notably, one study (Ur & Ormazá, 2012) investigated oral health practices and beliefs among informal and formal caregivers for the elderly. The results showed that formal caregivers were better educated in oral healthcare than ICs. Although the formal caregivers showed worse oral health practices than the ICs, these two groups of caregivers did not differ in their oral health beliefs. According to another study (Bonfa et al., 2017), caregivers positively perceived the oral health and self‐care actions that they provided to older people during home care.

Understanding ICs' perceptions of oral care is important because these views can be expected to heavily influence their personal oral hygiene, CRs' oral self‐care, and the use of oral health services. The aim of this study was to describe ICs' perceptions of oral care, as well as investigate whether these perceptions were associated with the use of oral health services during the past year among ICs and their CRs.

## 2 MATERIALS AND METHODS

### 2.1 Study design and sample

**Figure 1:**
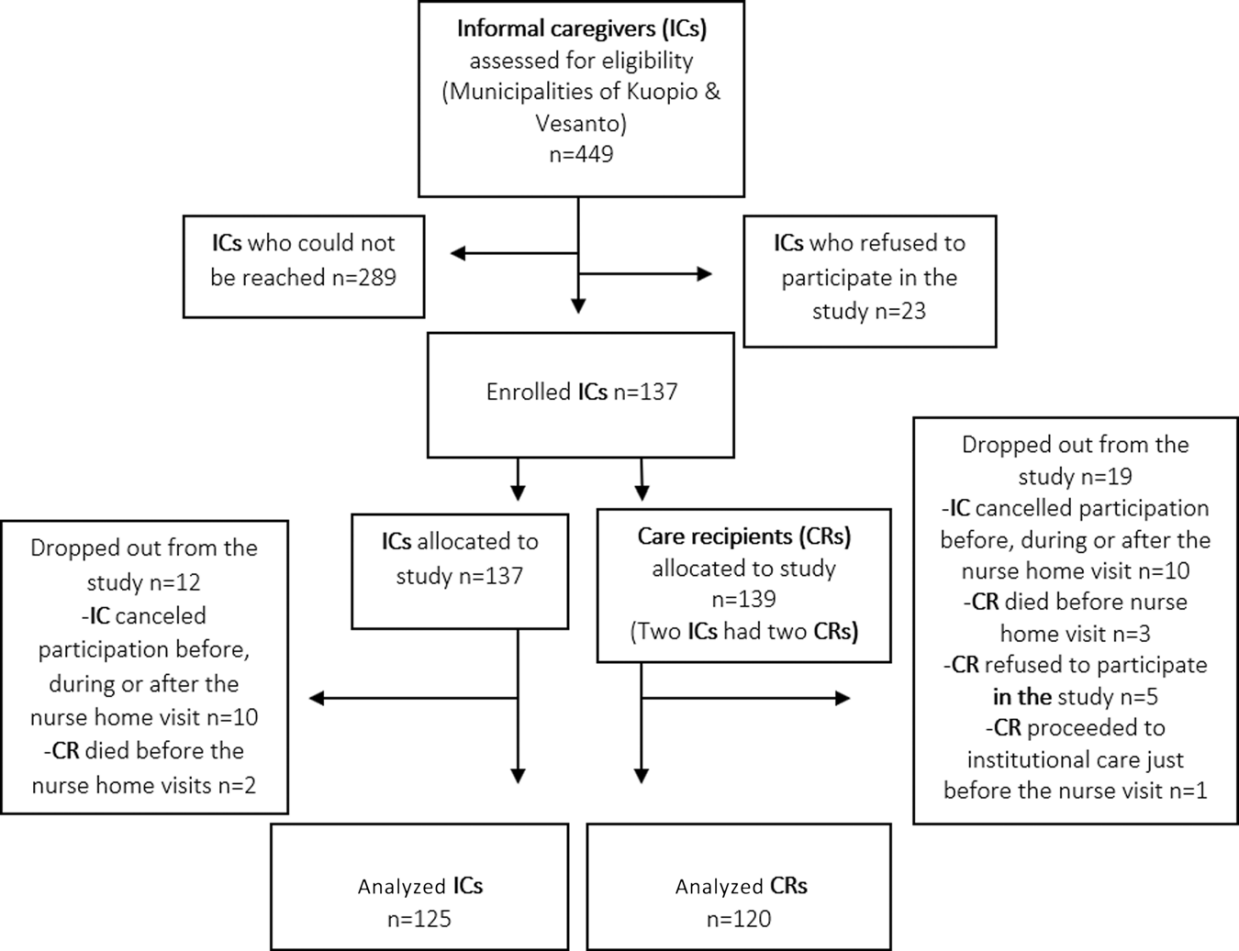
Sampling strategy and study sample

The baseline data used in this cross‐sectional study were from the multidisciplinary intervention study LENTO (Lifestyle, Nutrition, and Oral Health in Caregivers). The target groups consisted of all residents with informal care agreements in Kuopio and Vesanto in Eastern Finland. The criteria for participation in the study were: ICs had to have a valid informal care agreement; CR ≥ 65 years of age; CR not in terminal care; and CR living at home. In the city of Kuopio, the service manager sent invitation letters to ICs who were in the municipal IC register. The letter included information about the study and an invitation to participate. Those IC's who volunteered to participate contacted the research team. In the municipality of Vesanto, the service manager for older people contacted every IC in Vesanto. An invitation letter was first sent to all of the ICs who were interested in participating, after which the research team contacted the ICs who volunteered to take part in the study. The study group consisted of 125 ICs and 120 CRs. There were fewer CRs than ICs in the study because some of the CRs refused to participate, and one CR died before the first visit (although their IC still wanted to participate in the study). The flow chart of the study is presented in Figure 1.

### 2.2 Data collection

Data were collected through health and function questionnaires and semi‐structured interviews conducted by a trained dental hygienist and trained nurse and nutrition therapist at the participant's home. The nurse was the first to visit, with the dental hygienist and nutrition therapist visiting the week after. The dental hygienist interviewed the participants about oral care, oral health, and the use of oral health services. The questions included in the interview are listed in Table 1. In addition, the study nurse interviewed the ICs about sociodemographics, health, and functional ability. The collected sociodemographic information covered gender, age, and years of education. The participants were also asked about the duration of the IC's informal care agreement and their relationship to the CR.

**Table 1:** Dental hygienist interview questions

How important do you consider oral care to be?	a) Very important b) Quite important c) Somewhat important d) Not important at all
When did you visit oral health services last?	a) During the past year b) 1─3 years ago c) 4─5 years ago d) More than 5 years ago e) I have never visited oral health services
When you last visited oral health services, did you visit… (several answer options were allowed)	a) Dentist b) Dental hygienist c) Dental assistant d) Dental technician
Did the following factors prevent you from accessing the oral health services you wanted? (several answer options were allowed)	a) Waiting list b) Poor transportation connections c) High cost of care d) Dental fear e) Inappropriate behavior of an oral care professional f) Other:
Denture status…	a) Own teeth, no dentures b) Own teeth with dentures c) Full dentures d) Edentulous

Health and functional ability factors used in this study were measured through the Sense of Coherence (SOC‐13) scale, Geriatric depression (GDS‐15) scale, Activities of daily living (ADL) scale, and instrumental activities of daily living (IADL) scale.

The orientation to life questionnaire (SOC‐13 scale) focuses on a person's attitudes and measures how she/he responds to stressful life situations (Eriksson & Lindström, 2005; Lindblad et al., 2018). The 13‐item SOC questionnaire, which has a score range of 13–93, includes three components: comprehensibility (five items); manageability (four items); and meaningfulness (four items) (Holmefur et al., 2014; Saravia et al., 2015). High SOC scores indicate that a person is motivated and has the resources to cope with stressful and challenging life situations (European Council, 2016). The SOC score classification (low, medium, high) used in previous studies did not work in this study because of an oblique distribution of scores; for this reason, we used the median score (63) as the cut value in the SOC‐scale analysis, with scores ≥63 indicating high SOC. The GDS‐15 questionnaire evaluates depression among older people. In this questionnaire, a person will evaluate her/his depressive symptoms over the past week. The questions concern a person's everyday mood, attitudes, and feelings. This 15‐item questionnaire has a score range of 0‒15, with scores ≤4 indicating no depressive symptoms and a score of 15 indicating major depressive symptoms. GDS‐15 scores have been classified differently across numerous studies. In this study, the cut value was 5, with a score ≥5 indicating depressive symptoms.

The ADL and IADL questionnaires measure an older person's functional ability. The 10‐item ADL index (Barthel index) has a score range of 0‒100, and concerns the following activities: feeding; bathing; grooming; dressing; bowel and bladder control; toilet use; transfers (bed to chair and back); and mobility on level surfaces and stairs. The cut value for the Barthel index was 90, with a score ≤90 indicating dependency on other's help and a score between 91 and 100 indicating slight dependency or independency. The eight‐item IADL questionnaire has a score range of 0‒8, and concerns the following activities: the ability to use a phone; shopping; food preparation; housekeeping; doing laundry; using transportation; managing medications; and ability to handle finances. The cut value was 6, with scores ≤6 indicating dependency on other's help, and scores 7‒8 indicating independency.

### 2.3 Data analysis

Data were analyzed with IBM SPSS statistical software, version 27 (IBM Inc.). Descriptive statistics included cross‐tabulations of ICs' perceptions of oral care and ICs/CRs last visit to oral health services with sociodemographic, health, and functionality‐related factors. Differences in distributions were tested using the *χ* ^2^ test. Associations between ICs' perceptions of oral care and the use of oral health services during the past year were analyzed using binary logistic regression. The first regression model clarified how ICs' perceptions of oral care are associated with their use of oral health services during past year. The second regression model clarified how ICs' perceptions of oral care are associated with CRs' use of oral health services during the past year. The explanatory variables in these regression models were chosen based on previous research. In addition, the selection was guided by the available data. The ADL and IADL scales both measure functional ability, so the correlation between these scales was tested using Spearman's rho test (*ρ* = 0.231, *p* = .009). Despite low correlation, we chose to include one of these scales in the regression models to limit the number of predictors. We chose the ADL‐scale because it is better known and more commonly used than the IADL‐scale. The Omnibus *χ* ^2^ test was used to explore model fit and Nagelkerke's *R* ^2^ test was used to indicate the degree of explanation. A *p* < .05 was used as the threshold for statistical significance.

### 2.4 Ethical consideration

The Ethics Committee of the Hospital District of Northern Savo 171/2019 gave a favorable statement of the study protocol. Participation in the study was voluntary and the study participants provided written informed consent. The participants were also told that they have the opportunity to withdraw from the study at any time without explaining the reason. This study followed the principles of the General Data Protection Regulation (European Council, 2016).

## 3 RESULTS

### 3.1 Characteristics of ICs

Most (72%) of the ICs were females (Table 2). Over half of ICs had 10 years or more education. Over half (68%) of the ICs had had an informal care agreement in place for more than a year. In terms of relationship with the CR, most of the ICs were the spouse or common‐law partner (89%). Moreover, 12% of the participating ICs had full dentures and 50% of them were dentate without dentures. Slightly over half (56%) of the ICs had a low SOC, while a majority (78%) did not report depressive symptoms. Most ICs were independent or only needed slight help from others in daily activities, that is, 90% of the ICs showed an ADL score of 91 or higher while 85% showed IADL scores of 7–8 (85%).

**Table 2:** Characteristics of the participating informal caregivers (*n* = 125

	*n* (%)
Gender	
Male	35 (28)
Female	90 (72)
Age (years)	
≤64	12 (10)
65─84	100 (80)
≥85	13 (10)
Years of education	
≤9	46 (37)
≥10	79 (63)
Beginning of informal care agreement	
≤1 year	40 (32)
>Year ago	85 (68)
Informal caregiver relationship to the care recipient	
Spouse or common‐law partner	111 (89)
Daughter/son	12 (10)
Someone else	2 (1)
Denture status^a^	
Dentate, no dentures	63 (50)
Dentate, dentures	48 (38)
Full dentures/edentulous	14 (12)
SOC‐13 score^b^, ^c^	
13–62	69 (56)
63–91	54 (44)
GDS‐15 score^d^	
0–4	97 (78)
5–15	28 (22)
ADL score^e^	
0–90	13 (10)
91–100	112 (90)
IADL score^f^	
0–6	19 (15)
7–8	106 (85)

	Mean ± SD
SOC‐13 score^b^, ^c^	61.7 ± 6.7
GDS‐15 score^d^	3 ± 2.4
ADL score^e^	98 ± 3.5
IADL score^f^	7.8 ± 0.5

Abbreviations: ADL, activities of daily living; GDS, geriatric depression; IADL, instrumental activities of daily living; SOC, sense of coherence.

^a^ *n* = 124.

^b^ Sense of Coherence‐13 Scale: Scores ≥ 63 indicate high SOC. High SOC = person feels motivated, he/she has the resources to cope with difficult/stressful situations in life.

^c^ *n *= 123.

^d^ Geriatric Depression‐15 Scale: Scores ≥ 5 indicate depressive symptoms.

^e^ Activities of Daily Living Scale: Scores ≥ 91 indicate slight dependency on help from others or independence.

^f^ Instrumental Activities of Daily Living Scale: Scores 7–8 indicate independency.

### 3.2 Importance of oral care, visits, and perceived barriers to oral health services

A majority (81%) of the ICs considered oral care to be very important, with the remaining ICs (19%) considering oral care to be less important (Table 3). During the past year, 68% of the ICs and 46% of the CRs had used oral health services. During their last visit, 57% of ICs had visited the dentist, 25% had visited the dental hygienist, 9% had visited the dental assistant, and 9% had visited the dental technician. Correspondingly, 57% of CRs had visited the dentist on their last visit, with 23%, 14%, and 6% visiting a dental hygienist, technician, and assistant, respectively, during their last visit. The ICs felt that waiting lists (24%), high cost of care (13%), and poor transportation connections (4%) were the main barriers to receiving oral health services. A few (7%) of the ICs mentioned some other reason, namely, public oral health services do not work, poor physical ability, other diseases, rush in own life, laziness, or poor memory, as a barrier to receiving oral health services. Of the female ICs 87% and male ICs 66% considered oral care to be very important (*p* = .008; Table 4).

**Table 3:** Importance of oral care, barriers to receiving oral health services and time since last visit to oral health services (*n* = 125)

	*n* (%)
Importance of oral care (informal caregiver's perception)	
Very important	101 (81)
Less important	24 (19)
Barriers to receiving oral health services (informal caregiver's perception)^a^	
Waiting list	30 (24)
Poor transportation connections	5 (4)
High cost of care	16 (13)
Dental fear	4 (3)
Inappropriate behavior of an oral care professional	2 (2)
Some other reason	9 (7)
Last visit to oral health services (informal caregivers)	
During the past year	85 (68)
More than a year ago	40 (32)
Last visit to oral health services (care recipients)^b^	
During the past year	55 (46)
More than a year ago	65 (54)

^a^ Several answer options were allowed.

^b^ *n* = 120.

**Table 4:** Informal caregivers' perceptions of the importance of oral care, presented according to informal caregiver characteristics (*n* = 125)

	Importance of oral care		*p* Value
	*n* (%)		
Informal caregiver characteristics	Very important	Less important	
Gender			.008
Male	23 (66)	12 (34)	
Female	78 (87)	12 (13)	
Age (years)			.899
≤64	10 (83)	2 (17)	
65–84	80 (80)	20 (20)	
≥85	11 (85)	2 (15)	
Years of education			.582
≤9	36 (78)	10 (22)	
≥10	65 (82)	14 (18)	
Beginning of the informal care agreement			.413
≤1 year	34 (34)	67 (66)	
>Year ago	6 (25)	18 (75)	
Denture status^a^			.785
Dentate, no dentures	52 (84)	10 (16)	
Dentate, dentures	38 (79)	10 (21)	
Full dentures/edentulous	11 (79)	3 (21)	
SOC‐13 score^b^, ^c^			.245
13–62	53 (77)	10 (16)	
63–91	46 (85)	8 (15)	
GDS‐15 score^d^			.734
0–4	79 (81)	18 (19)	
5–15	22 (79)	6 (21)	
ADL score^e^			.708
0–90	10 (77)	3 (23)	
91–100	91 (81)	21 (19)	
IADL score^f^			.137
0–6	13 (68)	6 (32)	
7–8	88 (83)	18 (17)	

Abbreviations: ADL, activities of daily living; GDS, geriatric depression; IADL, instrumental activities of daily living; SOC, sense of coherence.

^a^ *n* = 124.

^b^ Sense of Coherence‐13 Scale: Scores ≥ 63 indicate high SOC. High SOC = person feels motivated, she/he has the resources to cope with difficult/stressful situations in life.

^c^ *n *= 123.

^d^ Geriatric Depression‐15 Scale: Scores ≥ 5 indicate depressive symptoms.

^e^ Activities of Daily Living Scale: Scores ≥ 91 indicate slight dependency on help from others or independence.

^f^ Instrumental Activities of Daily Living Scale: Scores 7–8 indicate independency.

### 3.3 Association between ICs characteristics and visits to oral health services during the past year

An IC's perception of the importance of oral care (*p* = .002) and that the high cost of care is a barrier to care (*p* = .005) significantly associated with their use of oral health services during the past year (Table 5). For example, 74% of the ICs who considered oral care to be very important had used oral health services during the past year. The corresponding proportion for ICs who considered oral care to be less important was 42%. Furthermore, 72% of ICs who did not feel that the high cost of care is a barrier to receiving oral care had used oral health services during the past year. The corresponding proportion for ICs who identified the high cost of care as a barrier to receiving oral health services was 38%. An IC's educational background demonstrated some association with their use of oral health services during the past year (*p* = .089). No association between IC characteristics and a CR's last visit to oral health services was observed.

**Table 5:** Informal caregivers' (*n* = 125) and care recipients' (*n* = 118) last visit to oral health services presented according to informal caregiver characteristics

	Time since last visit to oral health services					
	Informal caregiver			Care recipient		
Informal caregiver characteristics	During past year	More than a year ago		During past year	More than a year ago	
	*n* (%)	*n* (%)	*p* Value	*n* (%)	*n* (%)	*p* Value
Gender						
Male	20 (57)	15 (43)	.105	18 (51)	17 (49)	.496
Female	65 (72)	25 (28)		37 (45)	46 (55)	
Age (years)						
≤64	8 (67)	4 (33)	.767	5 (42)	7 (58)	
65–84	67 (67)	33 (33)		43 (46)	50 (54)	.820
≥85	10 (77)	3 (23)		7 (54)	6 (46)	
Years of education				*n* = 117		
≤9	27 (59)	19 (41)	.089	22 (49)	23 (51)	.747
≥10	53 (78)	15 (22)		33 (46)	39 (54)	
Beginning of the informal care agreement				*n* = 117		
≤1 year	27 (68)	13 (32)	.934	17 (46)	20 (54)	.876
>1 year ago	58 (68)	27 (32)		38 (48)	42 (52)	
Denture status	*n* = 124					
Dentate, no dentures	42 (68)	20 (32)	.687	31 (51)	30 (49)	
Dentate, dentures	32 (67)	16 (33)		20 (44)	25 (56)	.504
Full dentures/edentulous	11 (79)	3 (21)		4 (33)	8 (67)	
SOC‐13 score^a^	*n* = 123			*n* = 115		
13–62	48 (70)	21 (30)	.577	27 (44)	35 (56)	.428
63–91	35 (65)	19 (35)		27 (51)	26 (49)	
GDS‐15 score^b^				*n* = 117		
0–4	67 (69)	30 (31)	.632	44 (47)	49 (53)	.897
5–15	18 (64)	10 (36)		11 (46)	13 (54)	
ADL score^c^				*n* = 117		
0–90	9 (70)	4 (30)	.920	5 (39)	8 (61)	.513
91–100	76 (68)	36 (32)		50 (48)	54 (52)	
IADL score^d^				*n* = 117		
0–6	11 (58)	8 (42)	.305	6 (35)	11 (65)	.295
7–8	74 (70)	32 (30)		49 (49)	51 (51)	
Importance of oral care				*n* = 117		
Very important	75 (74)	26 (26)	.002	44 (46)	52 (54)	.586
Less important	10 (42)	14 (58)		11 (52)	10 (48)	
*Barriers to receiving oral care*						
High cost of care						
No	79 (72)	30 (28)	.005	47 (46)	55 (54)	.770
Yes	6 (38)	10 (62)		8 (50)	8 (50)	
Poor transportation connections						
No	83 (69)	37 (31)	.171	54 (47)	61 (53)	.641
Yes	2 (40)	3 (60)		1 (33)	2 (67)	
Waiting list						
No	64 (67)	31 (33)	.788	39 (43)	51 (57)	.201
Yes	21 (70)	9 (30)		16 (57)	12 (43)	

*Note*: *p* Value based on *χ* ^2^ test.

Abbreviations: ADL, activities of daily living; GDS, geriatric depression; IADL, instrumental activities of daily living; SOC, sense of coherence.

^a^ Sense of Coherence‐13 Scale: Scores ≥ 63 indicate high SOC. High SOC = person feels motivated, she/he has resources to cope with difficult/stressful situations in life.

^b^ Geriatric Depression‐15 Scale: Scores ≥ 5 indicate depressive symptoms.

^c^ Activities of Daily Living Scale: Scores ≥ 91 indicate slight dependency on help from others or independence.

^d^ Instrumental Activities of Daily Living Scale: Scores 7–8 indicate independency.

According to logistic regression analyses (Table 6), the ICs who had 10 or more years of education (odds ratio [OR]: 0.4, confidence interval [CI]: 0.2–1.1, *p* = .063), considered oral care to be very important (OR: 5.3, CI: 1.8–15.8, *p* = .003), and did not consider oral health service fees to be too high (OR: 9.0, CI: 2.5–32.9, *p* = .001) were significantly more likely to have visited oral health services during the past year than ICs who had fewer than 10 years of education, considered oral care to be less important, and considered oral health service fees to be too high. No association between IC characteristics and a CR's visit to oral health services during past year was observed.

**Table 6:** Associations between informal caregivers' characteristics and informal caregivers' and care recipients' last visit to oral health services during the past year

Informal caregivers' characteristics	Model 1 (*n* = 122)		Model 2 (*n* = 115)	
	Informal caregivers		Care recipients	
	OR	95% CI	OR	95% CI
Gender (ref. male)				
Female	1.9	0.7–5.0	1.1	0.5–2.0
Age years (ref. ≥85)				
≤64	0.4	0.0–3.5	1.5	0.3–9.6
65–84	0.4	0.1–2.3	1.4	0.4–5.4
Education years (ref. ≥10)				
≤9	0.4	0.2–1.1	1.2	0.4–1.9
Beginning of the informal care agreement (ref. >year ago)				
≤1 year ago	0.9	0.3–2.6	1.2	0.5–2.8
Denture status (ref. full dentures/edentulous)				
Dentate, no dentures	0.6	0.1–3.0	0.4	0.1–1.5
Dentate and partial dentures	0.7	0.1–4.0	0.5	0.1–2.0
SOC‐13 score^a^ (ref. ≤62)				
≥63	0.6	0.2–1.5	0.7	0.3–1.6
GDS‐15 score^b^ (ref. ≥5)				
≤4	1.3	0.4–3.9	1.1	0.4–3.0
ADL score^c^ (ref. ≥91)				
≤90	2.7	0.5–14.3	1.6	0.4–6.3
The importance of oral care (ref. less important)				
Very important	5.3**	1.8–15.8	1.4	0.5–3.9
Barriers to receive oral health services				
High cost of care (ref. yes)				
No	9.0***	2.5–32.9	1.3	0.4–4.1
Poor transportation connections (ref. yes)				
No	3.2	0.5–22.7	0.5	0.0–6.7
Waiting list (ref. yes)				
No	0.7	0.2–2.1	1.7	0.7–4.4

*Note*: Dependent reference category more than year ago.

Abbreviations: ADL, activities of daily living; CI, confidence interval; GDS, geriatric depression; OR, odds ratio; SOC, sense of coherence.

^a^ SOC‐13‐scores ≥ 63 indicate high sense of coherence, High SOC=Person feels motivated, she/he has resources to cope with difficult/stressful situations in life.

^b^ GDS‐15‐scores ≥ 5 indicate depressive symptoms.

^c^ ADL scores ≥ 91 indicate slight dependency on help from others or independence.

** *p* < 0.01

*** *p* < 0.001.

## 4 DISCUSSION

A majority of the ICs with a higher proportion were females than males considered oral care to be very important, with the rest of the participating ICs considering oral care to be less important. Interestingly, two‐thirds of the ICs, but only half of the CRs, had visited oral health services during the past year. Favorable perceptions of oral care were associated with the frequency at which ICs visited oral health services during the past year; this association did not hold for the CRs.

Professional caregivers' oral care knowledge, attitudes, and practices in the care of older people have been investigated earlier. For example, Chebib et al. (2021) showed that professional caregivers are well aware of the importance of oral care and its influence on overall health. Cornejo‐Ovalle et al. (2013) studied the oral health competence of caregivers who supported institutionalized older adults. They found that most caregivers considered their own oral care important, with a smaller proportion considering oral care among CRs to be important. We also found that most ICs consider oral care important; however, our study question did not distinguish whether an IC perceived their personal oral care or the CR's oral care as important.

A high proportion of the participating ICs had visited oral health services during the past year. A previous study (Välimäki et al., 2020) revealed that ICs who care for demented CRs use medical services less frequently than the average population. More specifically, they do not utilize supportive services because they do not want to leave their CR in the responsibility of another caregiver. This has been thought to explain why ICs use health services less than the general population but was not observed in our study of oral health services. This may be because the study sample consisted of ICs who were healthy or more health‐conscious. In this study, the proportion of CRs who had used oral health services during the past year fell below the average rate in Finland for people ≥65 years of age (Suominen‐Taipale et al., 2000) but was at a similar level as was reported in the GeMS‐study (Komulainen et al., 2012), which included home‐dwelling older people ≥75 years of age. Between‐sample differences explain these discrepancies. For example, the national survey also included healthy participants whereas our sample consisted of CRs with poor health. In the GeMS‐study, the low frequency at which older people used oral health services was found to be associated with the participants' poor levels of functional ability and cognitive function.

According to the collected data, about half of the participating ICs had visited a dentist during their last oral health services visit, with one in five visiting a dental hygienist and one‐tenth visiting a dental assistant or dental technician. Similar results were noted for CRs, which means that experiences with various oral health professionals do not explain the difference in visit rates between ICs and CRs.

Favorable perceptions of oral care were positively associated with oral health services during the past year among ICs, but not among CRs. Furthermore, ICs who felt that oral care is very important were more likely to frequently visit oral health services than ICs who felt that oral care is less important. This is in accordance with earlier findings, that is, favorable perceptions of oral care have been observed to influence regular use of oral health services (Riley et al., 2006; Tennstedt et al., 1994; Ur & Ormazá, 2012). However, ICs' perceptions of oral care were not associated with the frequency at which CRs use oral health services. We postulated that the CRs' poor physical ability, multimorbidity, ICs' hectic life, and ICs' potential poor memory could have influenced—to some extent—the frequency at which CRs use oral health services. Similar barriers have also been identified in previous oral health service studies of older people and home care clients (Komulainen et al., 2012; Nitschke et al., 2001). This result is important to consider when developing oral health services and models of co‐operation between informal care and oral health services. For example, ICs and their CRs could be provided with easily accessible (i.e., at home) oral health services that the ICs could be informed about. The GeMS‐study (Komulainen et al., 2012) showed that one‐fourth of home‐dwelling older people choose a dentist's home visit, and this decision was explained by poor functional ability and impaired cognitive function. A recall system for CRs could also help improve CRs' access to oral health services. Moreover, Finnish adults use oral health services less frequently than adults in other Nordic countries with similar health care systems. An explanation for this result may be a higher proportion of edentulous people in older age groups in Finland relative to other Nordic countries (Suominen & Raittio, 2018). However, the results of this study suggest that favorable perceptions of oral care might contribute to more frequent use of oral health services among ICs. It would be important to transfer this dynamic to the population of Finnish CRs. This finding is relevant because a prior longitudinal study stated that adults' attitudes towards oral care have changed negatively between 2003 and 2013, with fewer and fewer people considering regular oral health services to be important (Edman et al., 2018).

Several previous studies have identified high service fees to be a barrier to oral health services (Bahadori et al., 2013; Marino & Giacaman, 2017; Suominen & Raittio, 2018; Suominen et al., 2017). This issue was also present in our results, as ICs who did not consider service fees to be too high were more likely to visit oral health services than ICs who felt that oral health services were expensive. This has also been shown in Finnish national surveys, that is, the cost of care was found to affect access to treatment and maintain inequality (Bahadori et al., 2013). In addition, the presented results demonstrate that educational level positively influences the frequency at which an IC visits oral health services during the past year. This is relevant because the educational level has been observed to contribute to the frequency of health care services use in both Finland (Suominen & Raittio, 2018; Suominen‐Taipale et al., 2004) and abroad (Marino & Giacaman, 2017; Stewart et al., 2002) as well as among older people (Nitschke et al., 2001).

We chose to study the IC perspective because these people are responsible for both their own oral care as well as the oral care of CRs. In this study, a majority of the ICs were female and, in most cases, the IC was the spouse or common‐law partner of the CR. This agrees with Finnish statistics from 2019 (Noro et al., 2018). We consider the various validated measures to be a strength of this study. For example, we applied a commonly used indicator, “visits during the past year,” when defining the time since last visit to oral health services. When interviewing the participants, we used semi‐structured interviews. Face‐to‐face interviews can be considered reliable in this kind of study. Moreover, it should be noted that participants could ask for clarification of questions and supplement their answers during the interviews. When discussing generalizability, it should be stated that the study participants came from one part of Finland (Eastern Finland, Northern Savo). This could limit the generalizability of the results; however, oral health services and informal care are similar throughout Finland, so regional differences should be quite small. Only baseline data were analyzed in this study; hence, longitudinal analyses and larger sample sizes could provide more insight into ICs' perceptions of their personal oral care and oral care among CRs, as well as how these perceptions are associated with the use of oral health services. The presented results also include some unanswered questions about CRs' barriers to oral health services; using a qualitative approach to study this aspect of oral health care could provide valuable new information. It is possible that the ICs in this study might have been characterized by better health and functional ability than average ICs, which may have affected ICs' positive perceptions of oral care and the high frequency at which they used these services.

## 5 CONCLUSIONS

This study presents new information about ICs' perceptions of oral care and how these perceptions are associated with oral health services, for which there was no previous research data. Our findings support the conclusions of previous studies in that favorable perceptions of oral care contribute to the use of oral health services during the past year. However, these perceptions did not influence CRs' use of oral health services. Supporting older people in challenging situations requires collaboration between various healthcare professionals and family members (Meurman et al., 2018). As such, knowledge about ICs' perceptions of oral care can help health care professionals and decision‐makers develop oral health services and improve their accessibility. The presented results can be utilized to develop informal care and oral health service operating models.

## AUTHOR CONTRIBUTIONS

*Conceptualization the study*: Anna L. Suominen and Johanna Lammintakanen. *Methodology*: Anna L. Suominen. *Recruitment of participants*: Sohvi Koponen, Irma Nykänen. *Data collection*: Roosa‐Maria Savela. *Data curation*: Irma Nykänen, Sohvi Koponen, and Roosa‐Maria Savela. *Formal analysis*: Karoliina Holmavuo, Anna L. Suominen, and Irma Nykänen. *Data interpreting*: Karoliina Holmavuo, Anna L. Suominen, and Johanna Lammintakanen, writing and original draft preparation Karoliina Holmavuo. *Writing – review and editing*: Johanna Lammintakanen, Anna L. Suominen, Tarja Välimäki, Irma Nykänen, Roosa‐Maria Savela, Sohvi Koponen, and Ursula Schwab. *Supervision*: Anna L. Suominen, Johanna Lammintakanen, and Ursula Schwab. *Project administration*: Ursula Schwab. *Funding acquisition*: Anna L. Suominen, Irma Nykänen, Tarja Välimäki, and Ursula Schwab. All authors have read and agreed to the final version of the manuscript.

## CONFLICTS OF INTEREST

Tarja Välimäki is a board member of Carers Finland. Carers Finland did not participate in this study (no involvement in study design, content, conducting, analysis, or manuscript). The funders had no role in the design of the study; in the collection, analyses, or interpretation of data; in the writing of the manuscript, or in the decision to publish the results.

## Data Availability

The data that support the findings of this study are available from the authors upon reasonable request.
